# Improving the power of gene set enrichment analyses

**DOI:** 10.1186/s12859-019-2850-1

**Published:** 2019-05-17

**Authors:** Joanna Roder, Benjamin Linstid, Carlos Oliveira

**Affiliations:** Biodesix Inc, 2970 Wilderness Pl, Ste100, Boulder, CO 80301 USA

**Keywords:** Enrichment analysis, Gene set enrichment analysis, Statistical power

## Abstract

**Background:**

Set enrichment methods are commonly used to analyze high-dimensional molecular data and gain biological insight into molecular or clinical phenotypes. One important category of analysis methods employs an enrichment score, which is created from ranked univariate correlations between phenotype and each molecular attribute. Estimates of the significance of the associations are determined via a null distribution generated from phenotype permutation. We investigate some statistical properties of this method and demonstrate how alternative assessments of enrichment can be used to increase the statistical power of such analyses to detect associations between phenotype and biological processes and pathways.

**Results:**

For this category of set enrichment analysis, the null distribution is largely independent of the number of samples with available molecular data. Hence, providing the sample cohort is not too small, we show that increased statistical power to identify associations between biological processes and phenotype can be achieved by splitting the cohort into two halves and using the average of the enrichment scores evaluated for each half as an alternative test statistic. Further, we demonstrate that this principle can be extended by averaging over multiple random splits of the cohort into halves. This enables the calculation of an enrichment statistic and associated *p* value of arbitrary precision, independent of the exact random splits used.

**Conclusions:**

It is possible to increase the statistical power of gene set enrichment analyses that employ enrichment scores created from running sums of univariate phenotype-attribute correlations and phenotype-permutation generated null distributions. This increase can be achieved by using alternative test statistics that average enrichment scores calculated for splits of the dataset. Apart from the special case of a close balance between up- and down-regulated genes within a gene set, statistical power can be improved, or at least maintained, by this method down to small sample sizes, where accurate assessment of univariate phenotype-gene correlations becomes unfeasible.

## Background

Set enrichment analysis has become an important element of the bioinformatics and biostatistics toolkit. Such analyses can provide insights into the fundamental biological processes underlying different molecular or clinically-defined phenotypes [1]. Suppose that a dataset is available in which p attributes (e.g. protein abundances, expressions of genes) are measured for N instances (samples), each of which has an associated continuous or categorical phenotype. Instead of carrying out p univariate analyses to evaluate the correlations between each individual attribute with the phenotype across the N instances, set enrichment seeks to identify a consistent pattern of increased or decreased correlations (an enrichment) within a subset of the p attributes compared with the remainder. Attribute subsets can be selected which contain attributes associated with particular biological processes or pathways of interest.

There are many incarnations of set enrichment analysis, which differ mainly in the methods used to assess enrichment and its significance. An overview and comparison of a multitude of approaches can be found in Ackermann et al. [2]. One class of set enrichment analysis methods uses an enrichment score (ES) to capture the differences of the individual attribute-phenotype correlations between the attribute subset and its complement. One commonly used enrichment score approach, gene set enrichment analysis (GSEA) [3, 4], ranks the univariate correlations between attributes and phenotype and defines an enrichment score in terms of extrema of a running sum constructed from the ordered ranks. The statistical significance of the association between attribute subset (gene set) and phenotype captured by the enrichment score is determined based on a null distribution of the ES generated by permuting the phenotype labels.

The power of analyses such as GSEA to detect an association with a particular attribute subset depends on: *i.* the number of attributes measured; *ii.* the number of attributes in the attribute subset and correlations between them; *iii.* The number of samples for which data is available; and *iv*. the metric used to assess the univariate attribute-phenotype correlations. Considerable research has been performed to better understand the limitations of GSEA and how the factors listed above impact its sensitivity and statistical power (e.g., [5–7]). In this paper, we explore the dependence of the statistical power of the GSEA approach on the number of samples in the cohort with available molecular data. We show that, while the distribution of ES narrows with increasing N, the null distribution generated by phenotype permutation does not. Hence, increasing the number of samples in the cohort does not give the same increase in statistical power with N commonly observed in other settings. As a corollary, we show that, as long as the cohorts are large enough, splitting the cohort into two distinct parts and using the average of the ESs from each part as an alternative statistic provides greater power to detect associations than using the conventional ES defined using the entire cohort. This approach produces an enrichment statistic, and hence enrichment *p* value, that depends on the particular split of the cohort into two parts. This potential disadvantage can be mitigated by randomly selecting multiple cohort splits and averaging the ES over these splits, as well as over the halves in a particular split. We show that this technique can produce a desired level of precision (in enrichment score metric and *p* value) independent of how the cohorts are split.

## Results

### mRNA expression data for patients with breast cancer

**Fig. 1:**
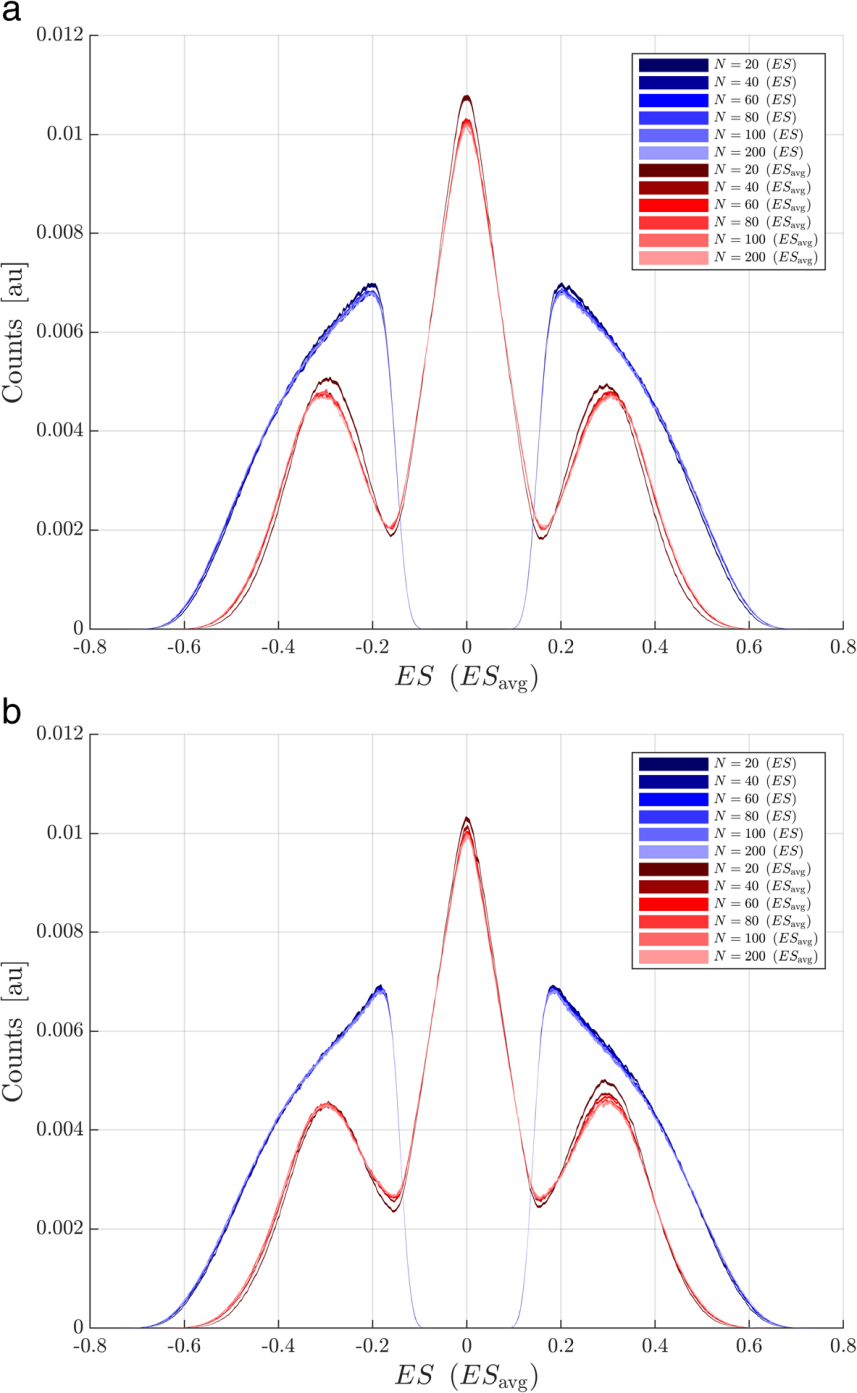
Null distribution for ES and ES_avg_ for N = 20, 40, 60, 80, 100, and 200. **a** HALLMARKS_MYC_TARGETS_V1, **b** HALLMARKS_ALLOGRAFT_REJECTION. Distributions for ES are shown in blue and those for ES_avg_ are shown in red

This section uses a publically available dataset with measurements of expression of 13,018 genes obtained from tissue samples collected from breast cancer patients. The cohort has been well-studied [8–10] and was the basis for development of a test stratifying patients into good or poor outcome groups following surgery for breast cancer [8, 9]. The test classifications (“good” or “poor”) are available as part of the dataset and are used as a binary phenotype. The data were accessed from the supplementary materials provided with Venet et al. [10]. The attribute subsets (here gene sets) used were the Hallmarks Gene Sets (a set of 50 gene sets) [11] available from the Broad Institute GSEA website (see Methods). Two particular gene sets, HALLMARK_MYC_TARGETS_V1 and HALLMARK_ALLOGRAFT_REJECTION, were chosen for particular investigation as examples of processes within the Hallmarks Gene Sets with association with phenotype within the breast cancer cohort characterized by GSEA *p* values of around 0.05 (*p* = 0.0172 for MYC_TARGETS_V1 and *p* = 0.0684 for ALLOGRAFT_REJECTION). The null distributions for the standard ES for the two gene sets are shown for various numbers of samples used in the enrichment analysis, N, in blue in Fig. 1a-b. The width of each band reflects the standard error of the null distribution in each histogram bin across the 1000 subset realizations created (random selections of N samples from the whole cohort, stratified by phenotype). It is apparent that the null distributions remain largely unchanged as N increases. Note that this contrasts with the archetypal, textbook case for typical statistics, e.g., Student’s t-statistic, where the null distribution narrows as N increases. The number of samples does not play a typical role in determining the width of the null distribution of ES. Other factors, such as number of attributes measured and number of attributes within the gene set, are much more important in determining the shape of the null distribution.

**Fig. 2:**
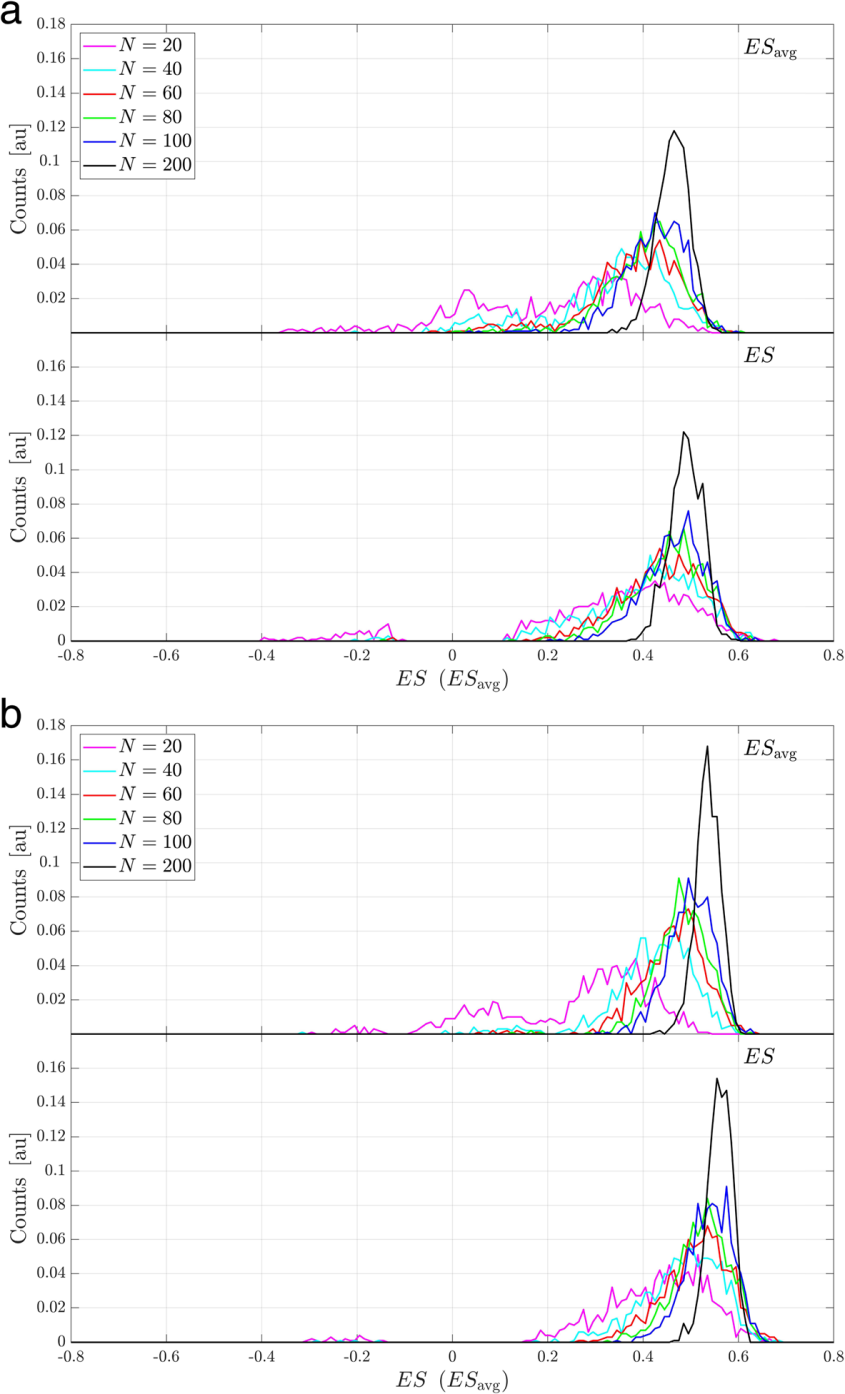
Sampling distribution for ES and ES_avg_ for *N* = 20, 40, 60, 80, 100, and 200. **a** HALLMARKS_MYC_TARGETS_V1, **b** HALLMARKS_ALLOGRAFT_REJECTION

For the same gene sets, the sampling distribution of ES, for subsets of N samples drawn from the studied cohort of 294 samples, does narrow as N increases (lower plots of Fig. 2a-b). For lowest N, the distribution retains a trace of the bimodal character of the null distribution. As N increases, the distribution becomes unimodal and then narrows further. Note that as sampling is performed within a population of only 294 samples, there will be correlations between sampling realizations, especially for larger N.

The results shown in Figs. 1 and 2 imply that the power to detect association between a particular attribute subset and phenotype will increase with N. However, it will not occur as quickly as for some simpler statistics, because although the distribution related to the alternative hypothesis narrows with N, the distribution for the null hypothesis does not.

We now consider the impact of changing the test statistic from the standard ES calculated using N samples to the average of the two ESs, ES1 and ES2, each calculated for a split of the N samples into two distinct subsets of N/2 samples, i.e. ES_avg_ = 0.5 (ES1 + ES2). Figure 1a-b compares the null distribution for ES_avg_ (in red) with that for ES (in blue) for various values of N for the two example gene sets. (Note that the null distribution of ES_avg_ is trimodal, not bimodal. For a permutation of phenotype classifications, ES1 and ES2 are equally likely to be positive or negative and hence it is not unlikely that ES_avg_ is close to 0.) Figure 2a-b shows the same for the sampling distributions of ES_avg_ (upper plots) and ES (lower plots). For all N studied, we observe that the null distribution for ES_avg_ is narrower than that for ES. This is a result of the relative independence from N of the null distributions: The null distribution of ES is similar for N and for N/2. So, the null distribution of ES1 and ES2 (which are calculated for N/2 samples) is similar to that of ES. As ES_avg_ is an average of ES1 and ES2, its null distribution for N samples will be narrower than those of ES (similarly ES1 and ES2) for N/2 samples, and hence be narrower than that of ES for N samples. For small N, the sampling distribution for ES_avg_ may be wider than that for ES. This occurs when N is so small that the phenotype-individual gene correlations cannot be evaluated with sufficient accuracy to produce a unimodal ES_avg_ sampling distribution, even though there is a true population association between gene set and phenotype. This can happen for larger N when there is no population association between gene set and phenotype. However, when there exists a true population association between gene set and phenotype, for larger N the sampling distribution for ES_avg_ for N samples is similar in location and width to that for ES. In these cases, illustrated by MYC_TARGETS_V1 and ALLOGRAFT_REJECTION, although the sampling distribution for ES1 and ES2 is broader than that for ES, due to the halving of the sample size, this is compensated for by the narrowing effect of averaging ES1 and ES2 together for the new statistic, ES_avg_.

**Fig. 3:**
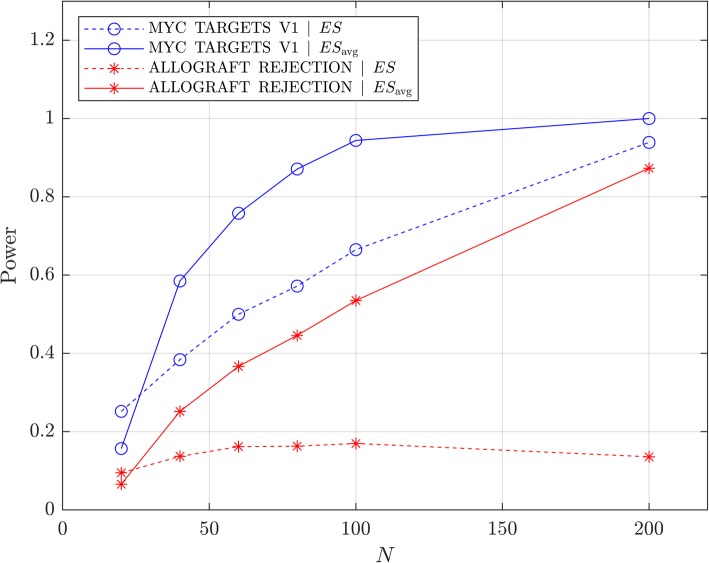
Power to detect association of phenotype with HALLMARKS_MYC_TARGETS_V1 (blue) and HALLMARKS_ALLOGRAFT_REJECTION (red) with α = 0.05. Power is shown as a function of N for ES (dotted line) and ES_avg_ (solid line)

Hence, using ES_avg_ as the test statistic increases the power of detecting the association of phenotype with a specific gene set over that obtained using ES, as long as N is not too small and there is a meaningful population association. Figure 3 shows the difference in statistical power between ES and ES_avg_ as test statistic to detect the association between the two example gene sets and phenotype. Results are shown as a function of subset size, N, of the 294 patient cohort. Even for 40 samples (24 “poor” and 16 “good” phenotype), using ES_avg_ as the statistic provides increased power to detect the association. For 20 samples, power is numerically smaller for the ES_avg_ than for ES, although both methods provide minimal power (less than 30%). The exact sample size at which benefit from ES_avg_ over ES ceases will depend on the magnitude of association. It is not possible to assess anything but very strong univariate correlations between phenotype and individual gene expression with any accuracy for very small sample sizes. In this setting, the power to detect the association of gene sets with phenotype using the standard ES test statistic is already severely impacted. This situation is exacerbated if the dataset is split in half. There will then be no improvement in power for ES_avg_ over ES, but the statistical power using either test statistic will be low.

**Fig. 4:**
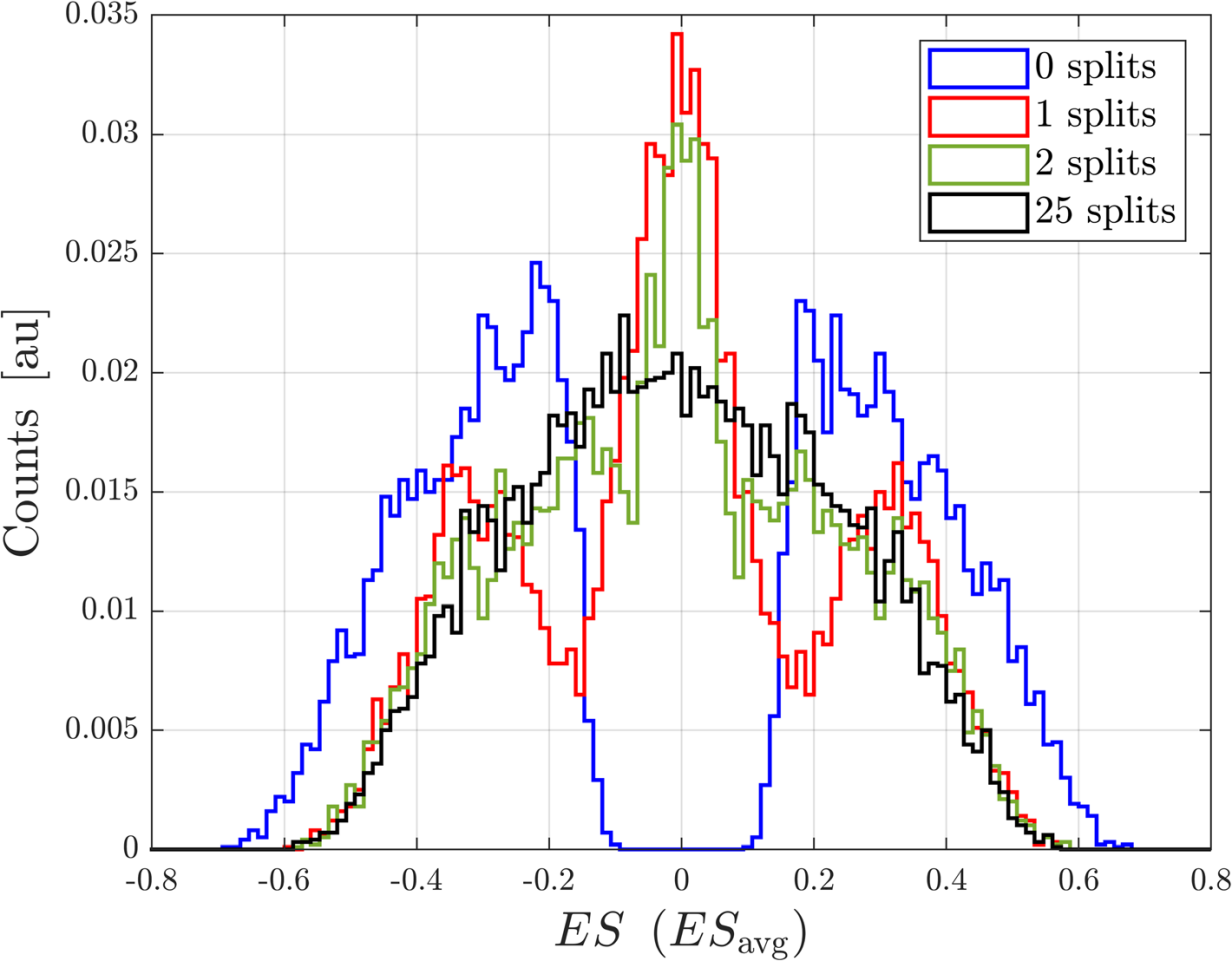
Null distributions for ES and for <ES_avg_>. Null distributions for<ES_avg_ > are shown for one split (ES_avg_ = <ES_avg_>), two splits, and 25 splits. All distributions are generated for one subset of 200 samples drawn from the 294-patient cohort

One disadvantage of using the statistic ES_avg_ is that it is not uniquely defined for a cohort and depends on the way that the cohort is split into two parts. This variability can be reduced by randomly splitting the cohort into two distinct parts many (M) times and defining a test statistic as the average of ES_avg_ over the M multiple splits, i.e. $<{\mathrm{ES}}_{\mathrm{avg}}>=\frac{\sum \limits_{splits}{\mathrm{ES}}_{\mathrm{avg}}}{\mathrm{M}}$. The appropriate null distribution can be generated by applying the same permutation of phenotype labels across all splits averaged for <ES_avg_>. Figure 4 shows the null distribution generated for one subset of *N* = 200 drawn from the cohort of 294 patients for the MYC_TARGETS_V1 gene set for a test statistic with no splits (ES), one split (ES_avg_), two splits and 25 splits of the subset. As the number of splits averaged increases above one, the distribution loses its multi-peak structure but retains the same overall width.

**Fig. 5:**
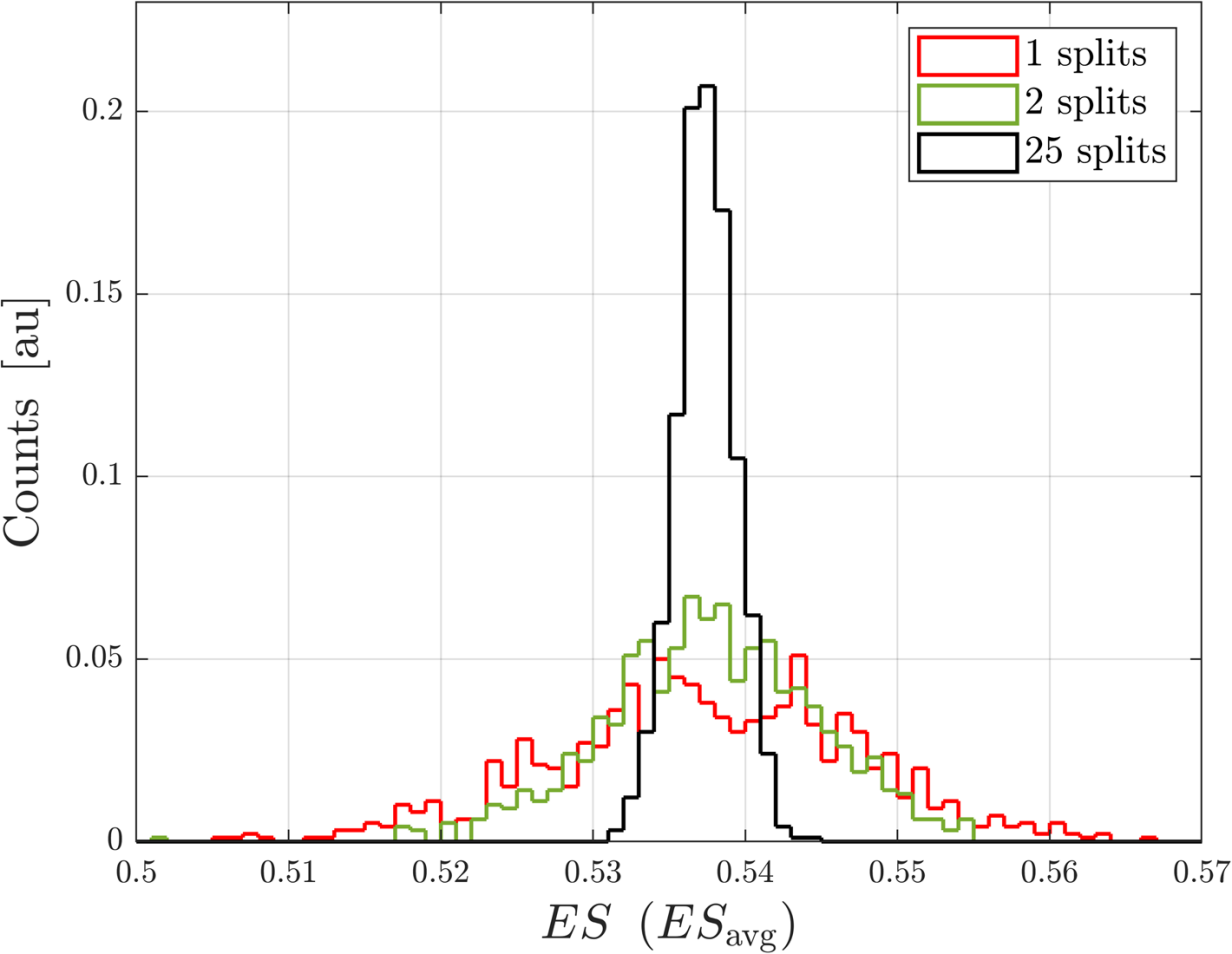
Distribution of ES_avg_, and < ES_avg_ > (two splits and 25 splits) for 1000 random splitting averages. All distributions are for a single subset of 200 samples using the MYC_TARGETS_V1 gene set

Figure 5 shows the distribution of the test statistics obtained for ES_avg_, and < ES_avg_ > for two splits and 25 splits for 1000 random splitting averages for the same single subset of 200 samples and the MYC_TARGETS_V1 gene set. As expected considering of the Law of Large Numbers, the location of the distribution remains unchanged and the width of the distribution narrows as the test statistic averages over more random splits. This procedure allows for definition of the test statistic, and hence associated enrichment *p* value, to arbitrary precision for the cohort by averaging sufficient random splits.

To illustrate the benefit of using ES_avg_ and < ES_avg_ > for 25 splits over ES as the test statistic over a wider range of gene sets, Table 1 compares the enrichment *p* values for all 50 Hallmarks Gene Sets as calculated using 294 patients using the three statistics. The *p* values of association are nearly always smaller for ES_avg_ and for < ES_avg_ > than for ES, and in the few cases where this is not the case, neither approach yields *p* values indicative of significant association.

**Table 1:** *p* values for the 50 Hallmarks gene sets. *p* values were calculated using the 294 sample cohort using ES, ES_avg_ or < ES_avg_ > with 25 splits as the test statistic. Gene sets are sorted by increasing *p* value obtained using ES_avg_ as the statistic

Gene Set	*p* value with ES	*p* value with ES_avg_	*p* value with <ES_avg_>
MTORC1_SIGNALING	< 0.0001	< 0.0001	< 0.0001
E2F_TARGETS	< 0.0001	< 0.0001	< 0.0001
UV_RESPONSE_UP	0.0132	< 0.0001	< 0.0001
G2M_CHECKPOINT	< 0.0001	< 0.0001	< 0.0001
PI3K_AKT_MTOR_SIGNALING	0.0040	0.0002	< 0.0001
MITOTIC_SPINDLE	0.0028	0.0004	< 0.0001
UNFOLDED_PROTEIN_RESPONSE	0.0006	0.0004	< 0.0001
REACTIVE_OXIGEN_SPECIES_PATHWAY	0.0063	0.0004	0.0002
ESTROGEN_RESPONSE_EARLY	0.0068	0.0006	0.0002
SPERMATOGENESIS	0.0185	0.0006	0.0002
GLYCOLYSIS	0.0216	0.0012	0.0008
MYC_TARGETS_V1	0.0172	0.0020	0.0002
UV_RESPONSE_DN	0.0156	0.0020	0.0012
MYC_TARGETS_V2	0.0320	0.0032	0.0026
DNA_REPAIR	0.0263	0.0035	0.0008
INTERFERON_GAMMA_RESPONSE	0.0373	0.0046	0.0038
IL6_JAK_STAT3_SIGNALING	0.0790	0.0074	0.0081
INTERFERON_ALPHA_RESPONSE	0.0638	0.0080	0.0105
COMPLEMENT	0.1059	0.0157	0.0149
ESTROGEN_RESPONSE_LATE	0.0622	0.0188	0.0080
ALLOGRAFT_REJECTION	0.0684	0.0194	0.0144
INFLAMMATORY_RESPONSE	0.0963	0.0303	0.0172
CHOLESTEROL_HOMEOSTASIS	0.1035	0.0449	0.0252
BILE_ACID_METABOLISM	0.0966	0.0472	0.0247
ANGIOGENESIS	0.2591	0.0796	0.0753
WNT_BETA_CATENIN_SIGNALING	0.4422	0.1160	0.1235
EPITHELIAL_MESENCHYMAL_TRANSITION	0.2984	0.1219	0.0984
COAGULATION	0.2516	0.1223	0.1093
IL2_STAT5_SIGNALING	0.1685	0.1437	0.0596
MYOGENESIS	0.2767	0.1589	0.1043
TGF_BETA_SIGNALING	0.3229	0.1593	0.1344
OXIDATIVE_PHOSPHORYLATION	0.3773	0.1877	0.1604
PROTEIN_SECRETION	0.3107	0.2032	0.2028
ADIPOGENESIS	0.4204	0.2247	0.2581
APICAL_SURFACE	0.4078	0.2477	0.0824
P53_PATHWAY	0.5724	0.2489	0.2423
TNFA_SIGNALING_VIA_NFKB	0.3401	0.2509	0.1545
HYPOXIA	0.4398	0.2712	0.2450
APOPTOSIS	0.5796	0.2905	0.3886
APICAL_JUNCTION	0.5175	0.2907	0.2579
NOTCH_SIGNALING	0.7451	0.3104	0.3226
FATTY_ACID_METABOLISM	0.5358	0.3134	0.3853
PANCREAS_BETA_CELLS	0.6834	0.3201	0.1500
XENOBIOTIC_METABOLISM	0.4921	0.3541	0.4946
HEME_METABOLISM	0.7713	0.4576	0.4731
KRAS_SIGNALING_UP	0.6241	0.7068	0.4892
ANDROGEN_RESPONSE	0.8082	0.7539	0.5841
HEDGEHOG_SIGNALING	0.7870	0.7810	0.5163
PEROXISOME	0.3931	0.8977	0.3682
KRAS_SIGNALING_DN	0.9700	0.9193	0.7337

### Synthetic dataset

To further investigate the performance of the method for attribute subsets with different levels of phenotype association and different degrees of attribute correlation, we carried out a set of experiments using synthetic data. Our approach is similar to the benchmarking methodology of Ackermann and Strimmer [2]. We simulated datasets of 600 genes for 50 samples (25 per phenotype) and defined 21 gene sets with differing degrees of inter-gene correlation and differential expression between phenotypes. Full details are provided in the Methods. To assess the power of the different test statistics to identify associations of phenotype with gene sets, we evaluated the proportion of the 100 dataset realizations in which an association was detected with *p* < 0.05 using ES, ES_avg_, and < ES_avg_ > for 25 splits. The results are shown in Table 2.

**Table 2:** Proportion of realizations with p < 0.05 for ES, ES_avg_, and < ES_avg_ > for 25 splits. The proportion was calculated over 100 realizations of the dataset for each of the 21 gene sets using the 3 test statistics, ES, ES_avg_, and < ES_avg_ > with M = 25. ^a^ indicates a control gene set with no association with phenotype

Gene Set	Proportion with *p* < 0.05		
	ES	ES_avg_	<ES_avg_>
a ^a^	0.06	0.08	0.05
b	1.00	1.00	1.00
c	0.82	0.81	0.92
d	0.09	0.09	0.14
e	0.38	0.39	0.46
f	0.06	0.13	0.10
g	0.01	0.00	0.01
h	0.29	0.19	0.19
i	0.10	0.16	0.07
j ^a^	0.07	0.07	0.07
k	0.92	0.93	0.98
l	0.81	0.88	0.91
m	0.92	0.94	0.98
n	0.34	0.35	0.43
o	0.73	0.76	0.84
p	0.42	0.56	0.64
q	0.77	0.84	0.90
r	0.22	0.26	0.22
s	0.75	0.77	0.90
t	0.36	0.38	0.44
u	0.25	0.28	0.37

With the exception of the two control sets (a and j), all gene sets are constructed with an association between at least some of the attributes in the gene set and the phenotype. The association is chosen to vary from moderate to weak. This allows for detection of differences in statistical power to identify association between gene set and phenotype; if associations were strong (e.g., greater than for gene set b), they would be uniformly detected in almost all realizations for all methods. For the two control gene sets, with no association between phenotype and gene set, the distribution of *p* values over the realizations was uniform (see histograms in Appendix) and the proportion of realizations yielding a *p* value of association below 0.05 remains around 5% for our approach. For the majority of other gene sets, the proportion of realizations identifying the association with *p* < 0.05 is higher for <ES_avg_ > (M = 25), and often also for ES_avg_, than for ES. This indicates increased power to identify the constructed associations over a variety of attribute subset scenarios, including different magnitudes of univariate association between phenotype and genes, mixtures of up- and down-regulated genes between phenotypes, and differences in correlation structure within the gene set. Apart from the controls, there are two other situations where increased power is not observed. The first includes those gene sets where the association is very weak (gene sets d, f, and g). All three test statistics have similarly poor power to identify very weak associations constructed between phenotype and gene set. The second situation includes special cases of balance between up- and down-regulated attributes within a gene set (gene sets h and i). Gene sets h and i are constructed with equal numbers of phenotypically up- and down-regulated attributes, all with exactly the same strength of univariate correlation with phenotype. In this very special setting, for any particular realization of the dataset, one is equally likely to calculate either a positive ES or a negative ES. For gene set h, *p* < 0.05 is found in around 30% of cases, but around half of these correspond to a positive ES and the other half to negative ES. When the dataset is split into two to calculate ES_avg_ and < ES_avg_>, each half is equally likely to yield a positive or negative ES, due to the exact balance between up- and down-association with phenotype. Averaging over this bimodal distribution yields a distribution centered around ES_avg_ = 0 or < ES_avg_ > =0 and hence a reduction in the power to identify a significant association between phenotype and gene set. Therefore, in this special setting of balance between extent and number of features with up- and down-association with phenotype, performance of the ES_avg_ and < ES_avg_ > test statistics is inferior to that of ES. However, as long as one is not close to a precisely matched scenario of up- and down-regulation, ES_avg_ and < ES_avg_ > show at least similar power to ES (see gene set r, with 13 genes with ∆μ = 0.5 and 7 with ∆μ = − 0.5) or greater power (gene sets l, p, and q, each with 15 genes with ∆μ = 0.5 and 5 with ∆μ = − 0.5). In a real world setting, very close balance in number and magnitude of opposing directions of differential gene expression between phenotypes is unlikely to occur within a gene set. Hence, the analyses of the synthetic data indicate that use of ES_avg_ or < ES_avg_ > is likely to increase power to detect associations with biological processes represented by the gene sets as long as the sample set size and strength of association is large enough to provide some minimal power for identification via the standard ES approach.

## Discussion and conclusions

The null distribution of the enrichment score, as defined in the GSEA approach to set enrichment analysis, is largely independent of the number of samples used within the analysis. Hence, increasing the sample cohort size, N, can only lead to increases in power to detect association between a gene set and a phenotype by narrowing the sampling distribution of ES. Splitting the cohort into two distinct equal parts, calculating the ES for each part, and averaging these to create a new test statistic, ES_avg_, can produce a markedly narrower null distribution and similar sampling distribution of ES. This approach leads to increased statistical power to detect significant associations between phenotype and attribute subset. In the majority of cases where this is not the case, neither ES nor ES_avg_ as test statistic leads to identification of significant association of phenotype and gene set, because no association exists, the attribute subsets are not strongly enough associated with phenotype for detection, or N is too small to allow meaningful assessment of correlations between individual genes and phenotype. In exceptional situations of close matching between number and magnitude of up- and down- regulated attributes between phenotypes, the sampling distribution of the ES statistic has the unusual property of being bimodal even for the largest sample sizes. Using ES_avg_ as test statistic can then reduce the power to identify associations. However, this situation is unlikely to occur outside synthetically produced datasets, and such scenarios could be identified by inspection of the running sum from which ES is calculated. (Similar magnitudes for the maximal and minimal deviation of the running sum from zero would be observed, even though the *p* value associated these values of ES would be small.) Unacceptable dependence of the test statistic and enrichment *p* value on the way the cohort is split to produce ES_avg_ can be avoided by using an extension of the averaging process to include multiple random splits of the cohort in the test statistic <ES_avg_ > .

Application of this approach could lead to clear advantages in the statistical power available to identify associations between biological processes or pathways and sample/patient phenotypes in all but the smallest sample cohorts, where the standard method also has very limited power. This may help to alleviate the issue of comparative reduced power for these kinds of ESs that has been pointed out in the literature [2]. Increased power would enable the reliable identification of weaker associations and increased certainty for identifications that may have borderline significance in terms of *p*-value and false discovery rate with the standard statistic. The method has been illustrated using a binary phenotype classification and one choice of phenotype-individual gene correlation metric, but it should be applicable to enrichment analyses using other correlation metrics or continuous phenotype scores. The benefit of using ES_avg_ or < ES_avg_ > over ES depends on the relative independence of the null distribution of ES on the number of samples, N. This phenomenon is a result of the way that the enrichment is assessed, via the extrema of the running sum (created from ranking and combining the attribute-phenotype correlations) and the generation of the null distribution via phenotype permutation. Each phenotype permutation for generation of the null distribution leads to a randomization of the values and rankings of the attribute-phenotype correlations. Hence, the manner in which the correlation between attribute and phenotype is evaluated should not be important, and our method should be directly applicable to GSEAs employing other correlation metrics (e.g. Spearman/Pearson r for continuous attributes).

Here, we explored only a split of the sample set into two distinct, equal parts. The method could be extended to average over splits of the dataset into more than two parts, and this should lead to improved performance by further narrowing of the associated null distribution. However, the benefit of splitting into more distinct subsets would require larger cohort sizes. The concept of averaging ESs across distinct subsets may also be useful to allow the combination of data from multiple cohorts of samples with identical available attributes. This could be especially useful if batch effects preclude merging of the multiple sample sets into a single cohort. Use of normalized ESs [4, 12] would also permit the same approach to be used to combine data from different cohorts of patients with different attributes available per cohort, even, for example, to combine genomic and proteomic panel data, provided that consistent phenotypes could be assigned to the multiple cohorts. Extending to the case of multiple data sources for a single cohort of patients would also be possible using an averaging over the ESs calculated per data source, provided that the null distribution was generated using a permutation of patient-defined phenotype class labels.

## Methods

### Dataset and gene sets: mRNA expression

The dataset used in this part of the study, accessed from [10], includes mRNA expression measurements of 13,018 genes from tissue samples collected from patients undergoing surgery for breast cancer. This cohort of 295 patients was the basis for development of a test stratifying patients into “good” and “poor” outcome groups [8, 9]. The test classification for each patient is included in the dataset and this binary result was used as the phenotype for which association with biological processes was sought. Gene expression values were used as in [10] without further processing or normalization. We used data from 294 of the 295 patients (data from sample NKI373 was not used) throughout our studies to allow splitting of the cohort into two distinct, equally-sized subgroups.

The attribute sets, in this case gene sets, used here are the Hallmarks Gene Sets [11] available from the Broad Institute GSEA website (http://software.broadinstitute.org/gsea/msigdb/collections/jsp#H). They are a well-curated collection of gene sets representing clearly defined biological states and processes. Fifty gene sets are included in the collection. For most of the analyses we selected two particular gene sets from the Hallmarks set, MYC_TARGETS_V1 and ALLOGRAFT_REJECTION, as examples. The test classification phenotype showed unambiguous, but not extreme, associations with these gene sets and, as such, they were considered to be particularly illustrative examples. *P* values for enrichment were also calculated for all 50 gene sets in the Hallmarks collection using ES, ES_avg_, and < ES_avg_ > (25 splits) as test statistics using data from all 294 samples.

### Dataset and gene sets: synthetic data

To investigate the dependence of the performance of the method on level of association and degree of correlation between attributes in the attribute subsets in a more controlled way, we carried out a set of analyses using synthetic datasets and attribute subsets, following the benchmarking approach of Ackermann and Strimmer [2].

A synthetic dataset of expression values for 600 attributes (genes) was generated by drawing from a multivariate normal distribution with unit variance for 25 samples with phenotype A and 25 samples with phenotype B. For attribute *i*, we define the difference in mean attribute value between A and B as ∆μ_i_. The correlation between attribute *i* and attribute *j* is defined as ρ_ij_. The 600 attributes were selected for the 50 samples as follows:

i. 420 with ∆μ = 0 and ρ = 0,
ii. 20 with ∆μ = 0.5 and ρ = 0,
iii. 20 with ∆μ = 0.25 and ρ = 0,
iv. 20 with ∆μ = 0.1 and ρ = 0,
v. 20 with ∆μ = 0.5 and ρ = 0.6,
vi. 20 with ∆μ = 0.25 and ρ = 0.6,
vii. 20 with ∆μ = 0.1 and ρ = 0.6,
viii. 10 with ∆μ = + 0.5 and 10 with ∆μ = − 0.5, with ρ = 0.6 within each subgroup of 10 and ρ = − 0.6 between the subgroups,
ix. 10 with ∆μ = + 0.5 and 10 with ∆μ = − 0.5, with ρ = 0,
x. 20 with ∆μ = 0 and ρ = 0.6.

Twenty one gene sets with varying degrees of phenotype association and varying intercorrelation were created by taking the following attribute groups:

- 20 from (i)
- 20 from (ii)
- 20 from (iii)
- 20 from (iv)
- 20 from (v)
- 20 from (vi)
- 20 from (vii)
- 20 from (viii)
- 20 from (ix)
- 20 from (x)
- 10 from (ii) and 10 from (v)
- 10 from (ii), 5 + 5 from (viii) (5 ∆μ = 0.5 and 5 ∆μ = − 0.5)
- 20 from (ii), (iii) and (iv)
- 20 from (v), (vi) and (vii)
- 20 from (ii)-(vii)
- 10 from (ix) with ∆μ = + 0.5, 5 from (viii) with ∆μ = − 0.5, and 5 from (viii) with ∆μ = + 0.5 and ρ = 0.6
- 10 from (ii), 5 + 5 from (viii) (5 ∆μ = 0.5 and 5 ∆μ = − 0.5)
- 3 from (ii), 10 ∆μ = 0.5 from (ix) and 7 with ∆μ = − 0.5 with from (ix)
- 10 from (i) and 10 from (ii)
- 10 from (i) and 10 from (v)
- 8 from (i) and 12 from (ii)-(x)

### Gene set enrichment analysis implementation

The enrichment set analysis methodology used closely follows the approach of Subramanian et al. [4]. Rank-based correlation, in the form of a Mann-Whitney test statistic scaled to range from 1 to − 1, was used to characterize association between expression of individual attributes and the binary phenotype. For the standard gene set enrichment analyses, the enrichment score, ES, used was exactly as defined in Subramanian et al. with *p* = 1. The null distributions for assessment of statistical significance of enrichment were obtained by repeated random shuffling (permutations) of the phenotype classifications.

The alternative enrichment assessment method using ES_avg_ was implemented as follows. The cohort of size N was split into two equal and distinct subgroups, S_1_ and S_2_, each of size N/2. For each subgroup an enrichment score was calculated as described above, to yield ES1, ES2 for S_1_, S_2_ respectively. The alternative statistic ES_avg_ was defined as the average of the two subgroup enrichment scores, i.e. ES_avg_ = 0.5(ES1 + ES2). The null distribution was again calculated via permutation of phenotype classifications. The phenotype classifications were shuffled, then the dataset was split into two halves, S_1_ and S_2_. ES1 and ES2 were calculated within S_1_ and S_2_, respectively and averaged to give ES_avg_ for the permutation realization.

Assessment of enrichment using an average over multiple splits used the test statistic <ES_avg_ > = 0.5 Σ_i_ (ES1_i_ + ES2_i_)/M, where the sum runs over the number of splits, M, of the N samples into two random subsets, S_1i_ and S_2i_, which have enrichment scores ES1_i_ and ES2_i_, respectively. To generate the null distribution, the phenotype classifications were shuffled, and then the dataset was randomly split into two halves M times. <ES_avg_ > is then calculated for the permutation realization. This is repeated for the number of permutation realizations required to generate the null distribution.

### Null distributions for ES and ES_avg_

The null distributions for the standard enrichment score, ES, and the alternative statistic, ES_avg_, were generated for subsets of the cohort of size 20, 40, 60, 80, 100 and 200 for the gene sets MYC_TARGETS_V1 and ALLOGRAFT_REJECTION. In each case a subset was chosen randomly, stratified by phenotype classification. Phenotype classifications were randomly permutated 10,000 times in each case.

### Sampling distributions of ES and ES_avg_

The sampling distributions for subsets of size N drawn from the population cohort of 294 samples were generated for ES and ES_avg_ for the gene sets MYC_TARGETS_V1 and ALLOGRAFT_REJECTION for *N* = 20, 40, 60, 80, 100 and 200. One thousand subsets were chosen randomly for each subset size, stratified by phenotype classification.

### Comparison of power to detect associations between ES and ES_avg_

The power to detect association of the phenotype with the gene sets MYC_TARGETS_V1 and ALLOGRAFT_REJECTION was calculated as follows. The null distributions for ES and ES_avg_ for different subset sizes were first calculated as outlined above. ES and ES_avg_ were calculated as described above, for 1000 realizations of each subset size, for estimation of the sampling distributions. For each realization for each subset size, ES and ES_avg_ were compared with their respective null distributions to determine whether an association with *p* < 0.05 was observed. The power to detect this association with α = 0.05 was defined as the proportion of realizations for which *p* < 0.05.

### Null distribution for enrichment score statistics for different numbers of splits, M

The null distributions for ES (no splits), for ES_avg_ (1 split) and for <ES_avg_ > with 2 and with 25 splits of one subset of 200 samples drawn from the 294 patient cohort were estimated. Each null distribution was generated as described above from 10,000 permutations of the phenotype classifications.

### Distributions of <ES_avg_ > over different splits of the cohort for different numbers of splits, M

The distributions of ES (no splits), for ES_avg_ (1 splits) and for <ES_avg_ > with 2 and with 25 splits over different random splits of the single subset of 200 samples drawn from the study cohort were estimated using 1000 realizations of the sets of splits needed for each statistic.

### Associations of all 50 Hallmark gene sets with phenotype classification for the cohort

For each of the 50 Hallmark Gene Sets, GSEA was performed separately using ES, ES_avg_, and < ES_avg_ > with M = 25 splits on the whole cohort of 294 samples. The null distributions for each gene set were estimated by 10,000 phenotype classification permutations.

### Synthetic data analyses

For each of the 21 gene sets, GSEA was performed separately using ES, ES_avg_, and < ES_avg_ > with M = 25 splits for 100 realizations of the synthetic dataset. The null distributions were estimated by 10,000 phenotype classification permutations. The power of the analyses to detect association between gene set and phenotype for α = 0.05 (significance level of 95%) was estimated by calculating the proportion of realizations in which the enrichment *p* value was lower than 0.05. To examine the distribution of *p* values for the two control gene sets (a and j), GSEA was performed for the statistics ES, ES_avg_, and < ES_avg_ > with M = 25 for 1000 realizations of the dataset.

### Software

Software implementing the method presented in this study is available in the PSEABiodesix repository at https://bitbucket.org/PSEABiodesix/pseabiodesix.

## Figures and Tables

**Fig. 6:**
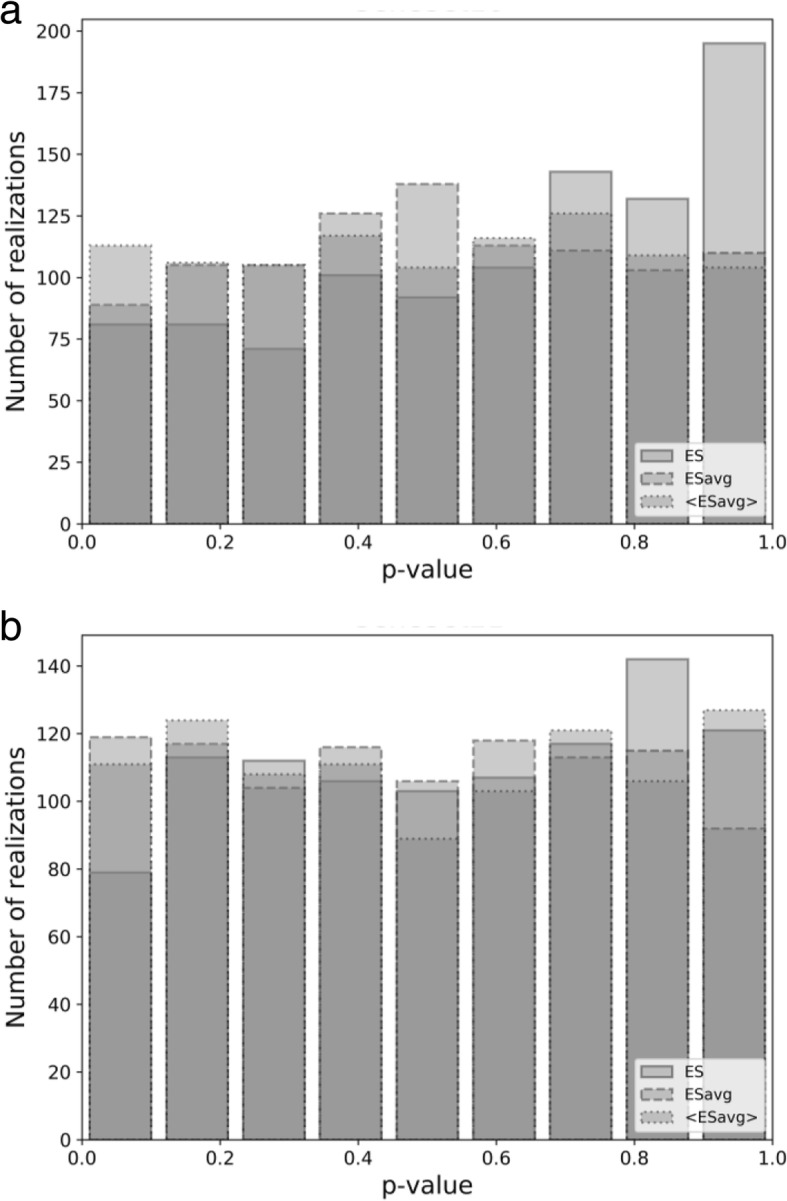
*p* value distributions over dataset realizations for ES, ES_avg_ and < ES_avg_ > for control gene sets. **a** Gene Set a, **b** Gene Set j

## Appendix

For the control synthetic gene sets, which had, by construction, no association with phenotype, GSEA was carried out using all three enrichment statistics on 1000 realizations of the dataset to examine the distribution of *p* values across the realizations (Fig. 6).

## Abbreviations

ES: Enrichment score
GSEA: Gene set enrichment analysis

## References

[1] Tilford CA, Siemers NO (2009). Gene set enrichment analysis. Methods Mol Biol.

[2] Ackermann M, Strimmer K (2009). A general modular framework for gene set enrichment analysis. BMC Bioinformatics.

[3] Mootha VK, Lindgren CM, Eriksson KF, Subramanian A, Sihag S, Lehar J, Puigserver P, Carlsson E, Ridderstrale M, Laurila E, Houstis N, Daly MJ, Patterson N, Mesirov JP, Golub TR, Tamayo P, Spiegelman B, Lander ES, Hirschhorn JN, Altshuler D, Groop LC (2003). PGC-I α-responsive genes involved in oxidative phosphorylation are coordinately downregulated in human diabetes. Nat Genet.

[4] Subramanian A, Tamayo P, Mootha VK, Mukherjee S, Ebert BL, Gilette MA, Paulovich A, Pomeroy SL, Golub TR, Lander ES, Mesirov JP (2005). Gene set enrichment analysis: a knowledge-based approach for interpreting genome-wide expression profiles. Proc Natl Acad Sci U S A.

[5] Tamayo P, Steinhardt G, Lizerzon A, Mesirov JP (2016). The limitations of simple gene set enrichment analysis assuming gene independence. Stat Methods Med Res.

[6] Zyla J, Marczyk M, Weiner J, Polanska J (2017). Ranking metrics in gene set enrichment analysis: do they matter?. BMC Boinformatics.

[7] Tarca AL, Bhatti G, Romero R (2014). A comparison of gene set analysis methods in terms of sensitivity, prioritization and specificity. PLoS One.

[8] van’t Veer LJ, Dai H, van de Vijver MJ, He YD, Hart AAM, Mao M, Peterse HL, van der Kooy K, Marton MJ, Witteveen AT, Schreiber GJ, Kerkhoven RM, Roberts C, Linsley PS, Bernards R, Friend SH (2002). Gene expression profiling predicts clinical outcome of breast cancer. Nature.

[9] van de Vijver MJ, He YD, van’t Veer L, Dai H, Hart AM, Voskuil DW, Schreiber GJ, Peterse JL, Roberts C, Marton MJ, Parrish M, Atsma D, Witteveen A, Glas A, Delahaye L, van der Velde T, Bartelink H, Rodenhuis S, Rutgers ET, Friend SH, Bernards R (2002). A gene expression Signature as a predictor of survival in breast cancer. New Engl J Med.

[10] Venet D, Dumont JE, Detours V (2011). Most random gene expression signatures are significantly associated with breast cancer outcome. PLoS Comput Biol.

[11] Liberzon A, Birger C, Thorvaldsdóttir H, Ghandi M, Mesirov JP, Tamayo P (2015). The molecular signatures database (MSigDB) hallmark gene set collection. Cell Syst.

[12] GSEA User Guide, http://software.broadinstitute.org/gsea/doc/GSEAUserGuideFrame.html. Accessed 6 Oct 2018.

